# A Prospective Cohort Study to Assess Neonatal Adaptation in Neonates Exposed to Psychotropic Medications in Utero

**DOI:** 10.1192/bjo.2022.221

**Published:** 2022-06-20

**Authors:** Firdous Murad

**Affiliations:** Irish College of Psychiatry, Ireland, Dublin, Ireland

## Abstract

**Aims:**

We aimed to compare the NAS in neonatal exposed to antidepressants to unexposed neonates.

**Methods:**

A prospective cohort study was carried out comprising of women in 3rd trimester of pregnancy, data were collected on women exposed and unexposed to antidepressants. Approval from the Rotunda Hospital REC was obtained.

Hospital records were used to collect pre-, peri- and postnatal information which was relevant to the study Aim. Neonatal Abstinence Score was completed within 0–48 hours of the birth, Moderate-Severe abstinence was defined as eight points or higher (on a scale with maximum 40 points), mild abstinence as 4 points or higher. Paediatric records were reviewed where the baby required NICU admission. Women were recruited between 2019–2021

**Results:**

221 women in total were recruited,138 pregnant women were on no psychotropic medication (Control group) and 83 pregnant women were on antidepressant medication (exposed group).

In the exposed group, 46% (38/83) were on Sertraline,19% (16/83) fluoxetine, 17% (14/83) Escitalopram and 17% (14/83) on other SSRI/SNRI.

Six infants (3%) expressed signs of severe abstinence and 38 (28%) had mild abstinence symptoms in exposed group whereas in control group 10 (7%) of infants were observed to have mild abstinence which was seen in infants with low birth weight, poor feeding and poor sleep after feed. Neonatal hypoglycaemia in infants prenatally exposed to antidepressant was seen in 10% compared to 1% of control group.

**Conclusion:**

Severe abstinence in infants prenatally exposed to antidepressants was found to be (3%) and mild abstinence in 28% this is in keeping with international findings. Low one minute APGAR sores and greater rates of hypoglycaemia were also noted.

While neonatal withdrawal with all antidepressants are usually mild and self-limiting it is important to make the obstretric and neonatal teams aware of the mothers medication and mothers should be advised that their baby may need a review by neonatology after delivery.

